# Parathyroid adenoma detected with ^99m^Tc-tetrofosmin dual-phase scintigraphy: a case report

**DOI:** 10.1186/1756-0500-7-335

**Published:** 2014-06-04

**Authors:** Konstantinos Romanidis, Evangelos Karathanos, Eleni-Aikaterini Nagorni, Alexandra Giatromanolaki, Efthimios Sibridis, Athanassios Zissimopoulos, Theodosia Vogiatzaki, Constantinos Simopoulos, Michael Pitiakoudis

**Affiliations:** 1 Second Department of Surgery, Medical School, Democritus University of Thrace, Dragana, Alexandroupolis 68100, Greece; 2 Department of Nuclear Medicine, Medical School, Democritus University of Thrace, Dragana, Alexandroupolis 68100, Greece; 3 Department of Pathology, University General Hospital of Alexandroupolis, Alexandroupolis, Greece; 4 Department of Anaesthesiology, Democritus University of Thrace, Alexandroupolis, Greece

**Keywords:** Parathyroid adenoma, ^99m^Tc-tetrofosmin dual-phase scintigraphy, p-glycoprotein, Nuclear medicine, Parathormone, Parathyroidectomy

## Abstract

**Background:**

Tc-sestamibi is the most frequently used radionuclide agent for the detection of parathyroid adenomas in the clinical setting. However, Tc-tetrofosmin is another such agent that may be used for this purpose. This case report presents the significance and practicality of ^99m^Tc-tetrofosmin for the diagnosis of parathyroid adenomas with probable high p-glycoprotein levels.

**Case presentation:**

A 45-year-old woman was referred to our Nuclear Department with a palpable neck nodule suspicious for parathyroid adenoma. She had no significant medical history or other accompanying symptoms. Blood examination results were normal with the exception of the parathormone level which was high at 167.2 pg/ml. Neck ultrasonography revealed a hypoechoic mass near the lower pole of the thyroid gland. ^99m^ Tc-tetrofosmin dual-phase scintigraphy with early and delayed images was performed and the results supported the presupposition of parathyroid adenoma as shown by increased radiopharmaceutical levels near the lower left thyroid gland on the early image that disappeared on the delayed image. Moreover, TcO_4_^−^ thyroid scintigraphy results excluded thyroid pathology. Two months after the diagnosis, parathyroidectomy was successfully performed without postoperative complications. The pathology report and clinical response to a gradual decrease of parathormone confirmed the initial diagnosis.

**Conclusion:**

We strongly recommend the use of reverse ^99m^ Tc-tetrofosmin scintigraphy as a useful and safe examination technique for the diagnosis of parathyroid adenomas.

## Background

Parathyroid adenoma is a major cause of primary hyperparathyroidism with an incidence of 80% to 85% [1]. The average size and weight of a normal parathyroid gland is 5 × 3 × 1 mm and 40 to 50 mg, respectively. Thus, this gland is infrequently identified during imaging. Conversely, adenomas are considerably larger, and have a mean mass of more than 10 times the normal parathyroid gland, they are thus often identified during cross-sectional imaging. Ultrasonography and ^99m^Tc-sestamibi scintigraphy are the dominant imaging techniques for diagnosis and preoperative localization of parathyroid adenomas [2]. ^99m^Tc-sestamibi scintigraphy is the method of choice for the localization of parathyroid adenomas because of its high diagnostic sensitivity (>90%) [1]. Although ^99m^Tc- sestamibi is the technique of choice, scintigraphy with ^99m^Tc-tetrofosmin has also been used to detect parathyroid adenomas with great success [3,4].

We herein present a case of a woman with a parathyroid adenoma close to the lower left thyroid gland diagnosed with ^99m^Tc-tetrofosmin dual-phase scintigraphy.

## Case presentation

A 45-year-old Greek female patient with a palpable neck nodule was referred to the Nuclear Medicine Department to undergo evaluation for a possible parathyroid adenoma. During the physical examination, the neck nodule was palpable and painless and measured approximately 1 cm. Her medical and family histories were not significant.

**Figure 1:**
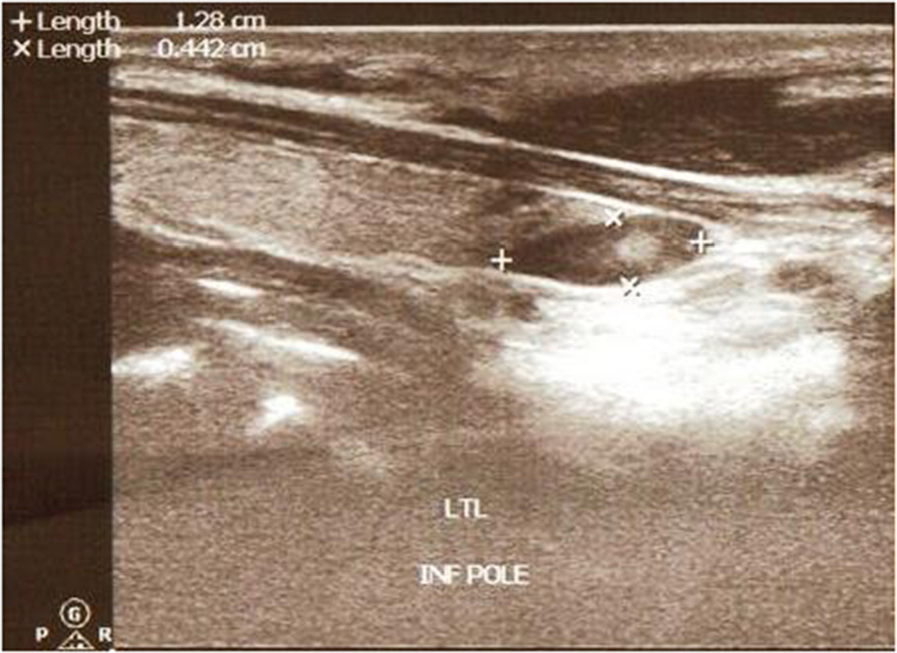
A neck ultrasound image showing a hypoechoic lesion (1.23 cm x 0.442 cm) close to the lower pole of thyroid gland.

A neck ultrasound had been performed two weeks earlier and had revealed a 1.203 x 0.442 cm hypoechoic mass suspected of being a parathyroid adenoma. This mass was located near the lower pole of the thyroid gland (Figure 1).

Blood examination showed an excessively high parathormone (PTH) level (167.2 pg/ml) (normal range: 15–65 pg/ml). However, other parameters were within their normal ranges (phosphorus: 2.8 mg/dl, calcium: 9.9 g/dl, thyroid-stimulating hormone :4,52 mU/l, free triiodothyronine (FT3):3.81 pg/ml, free thyroxine (FT4):8.27 pg/ml, 1,25(OH)2vitamin D:6 ng/ml, and alkaline phosphatase (ALP): 47U/L).

**Figure 2:**
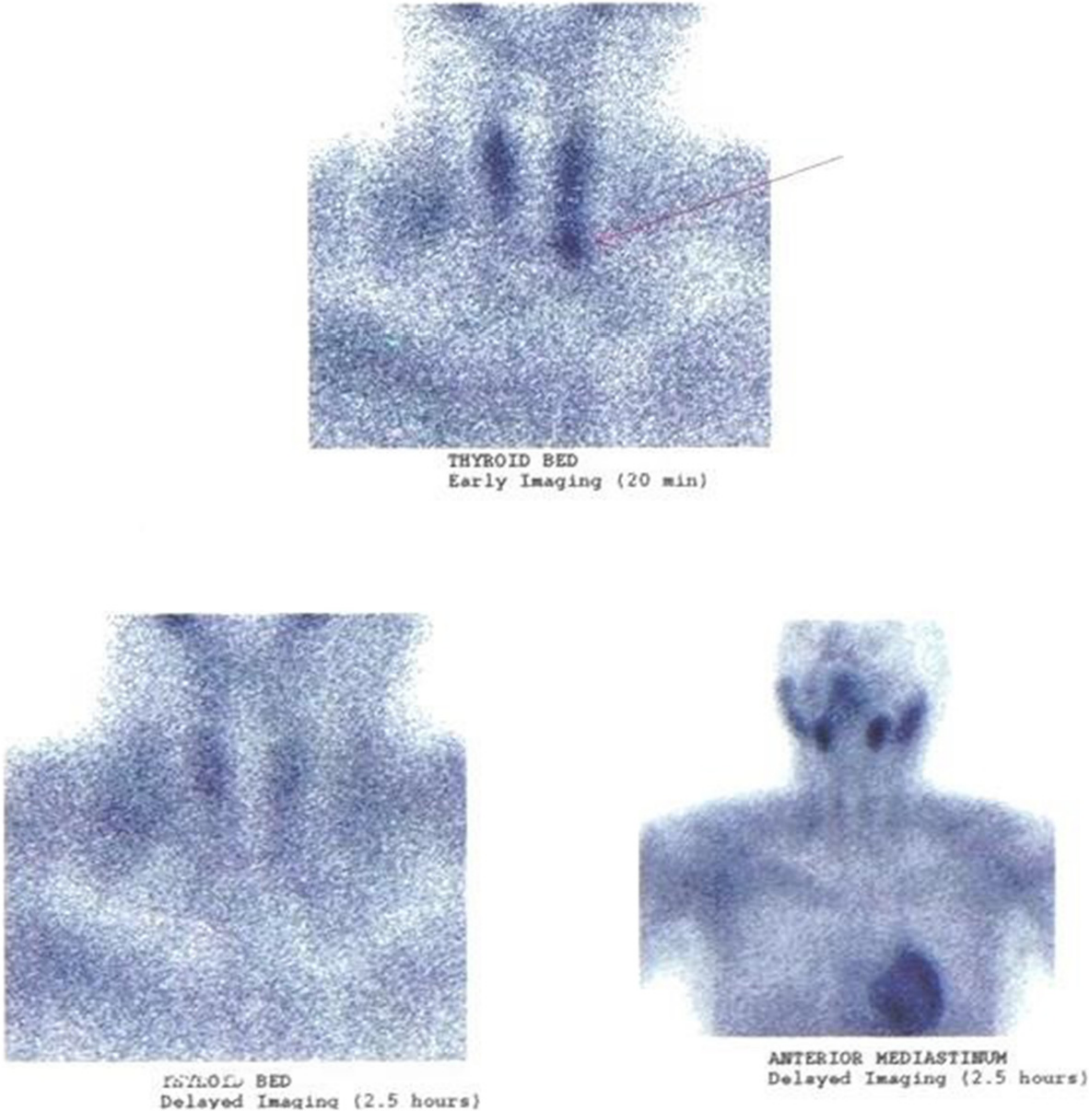
**Parathyroid scintigraphy with **^**99m**^**Tc-tetrofosmin.** Long legend: A focus of intense radiopharmaceutical retention in the thyroid bed during the early acquisition phase (at 20 minutes), just below to the lower pole of the left thyroid lobe. The late acquisition phase (at 2.5 hours) showed normal radiopharmaceutical wash-out from the whole thyroid gland, while the initial hot spot sign disappeared.

Dual-phase parathyroid scintigraphy technique was then performed, in which 20 mCi of ^99m^Tc-tetrofosmin was administered intravenously, followed by early images (20 minutes pi) and delayed images (2.5 hours pi) of the neck and the anterior mediastinum. Planar images of 100,000 counts were obtained on a large field of view camera at a 140 KeV photopeak with a 15% window and a low-energy high resolution collimator. Scintigraphy revealed a focus of intense radiopharmaceutical retention in the thyroid bed during the early acquisition phase (at 20 minutes), just below the lower pole of the left thyroid lobe. This sign corresponded to the sonographic finding. The late acquisition phase (at 2.5 hours) showed normal radiopharmaceutical wash-out from the whole thyroid gland, while the initial hot spot disappeared. Mediastinal imaging findings were normal with no detection of ectopic parathyroid glands. The above finding (intense focus on early acquisition and disappearance on late imaging) raised a strong suspicion of a left lower parathyroid adenoma, probably rich in high p-glycoprotein (p-gp) (Figure 2).

**Figure 3:**
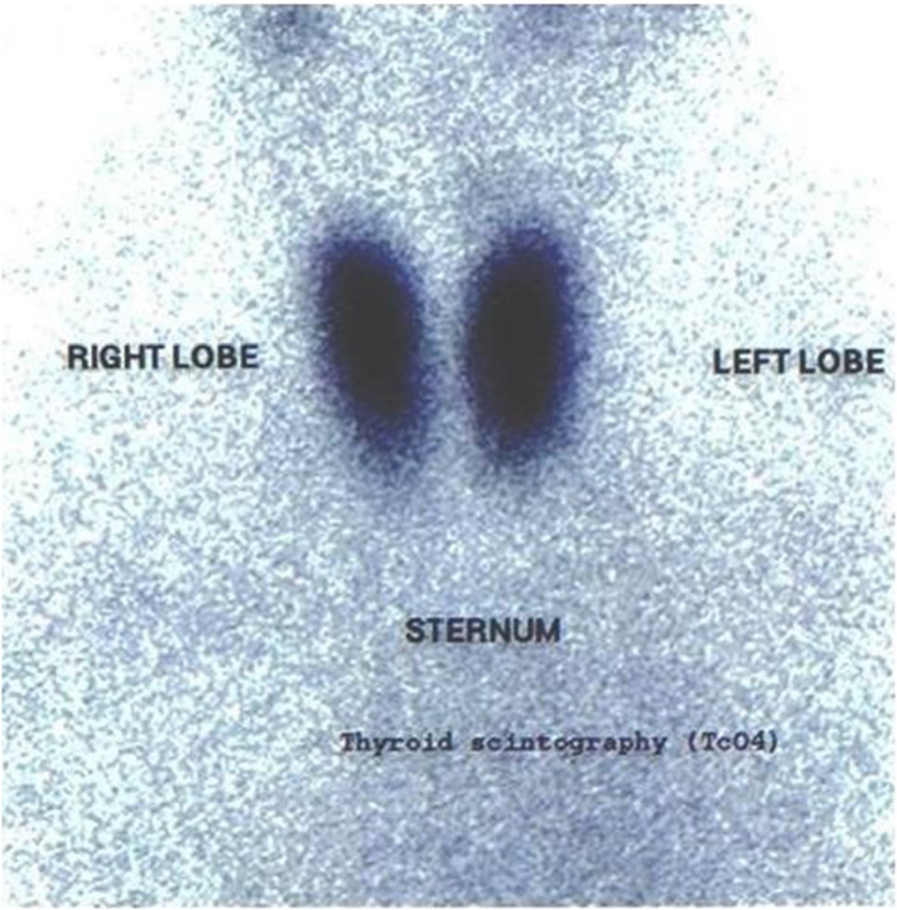
**Thyroid scintigraphy with 5 mCi TcO**_**4: **_**normal uptake of the tracer in the thyroid gland.**

To exclude thyroid pathology, thyroid scintigraphy with 5 mCi TcO_4_^−^ was carried out one day later. This study revealed normal uptake of the tracer in the thyroid gland. The intense focus seen on previous parathyroid imaging (as shown in the early phase at 20 minutes) was not depicted, supporting the diagnosis of a parathyroid adenoma on the above mentioned position (Figure 3).

Two months later, surgical resection of the parathyroid adenoma was successfully performed. Parathyroidectomy was performed under general anesthesia, followed by creation of a left transverse incision 3 cm from the mass; the procedure concluded with identification and removal of the left inferior parathyroid gland using ligatures.

**Figure 4:**
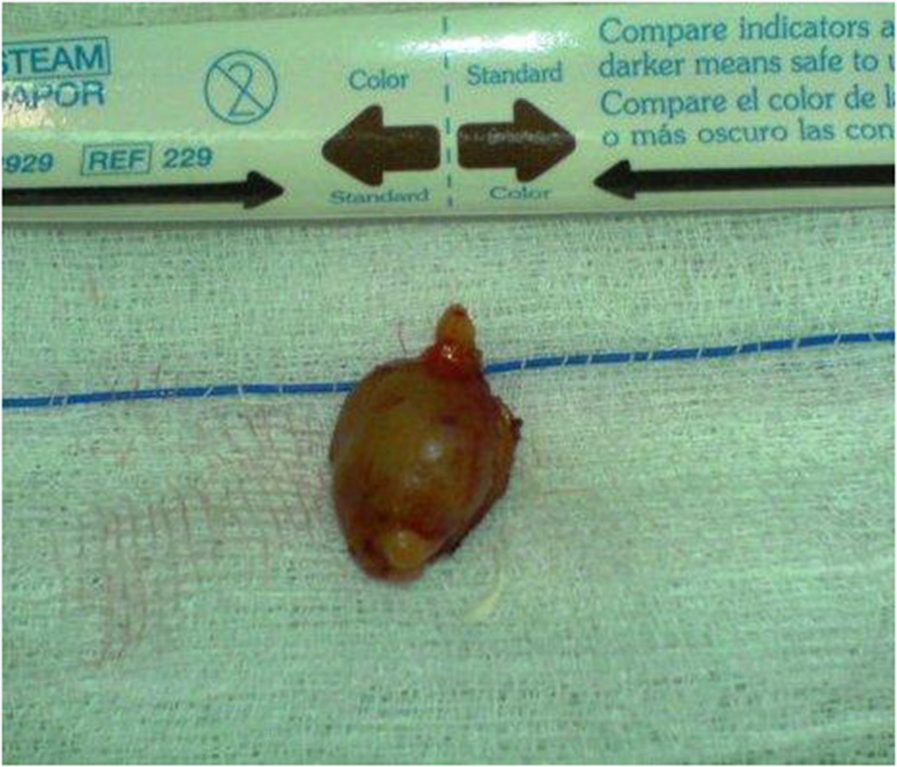
Macroscopical view of the left inferior parathyroid gland measuring 1 cm.

Macroscopically, the gland appeared as a solid, gray-brown, enlarged nodule with a maximum diameter of about 1 cm (Figure 4). The specimen was sent for pathology examination.

**Figure 5:**
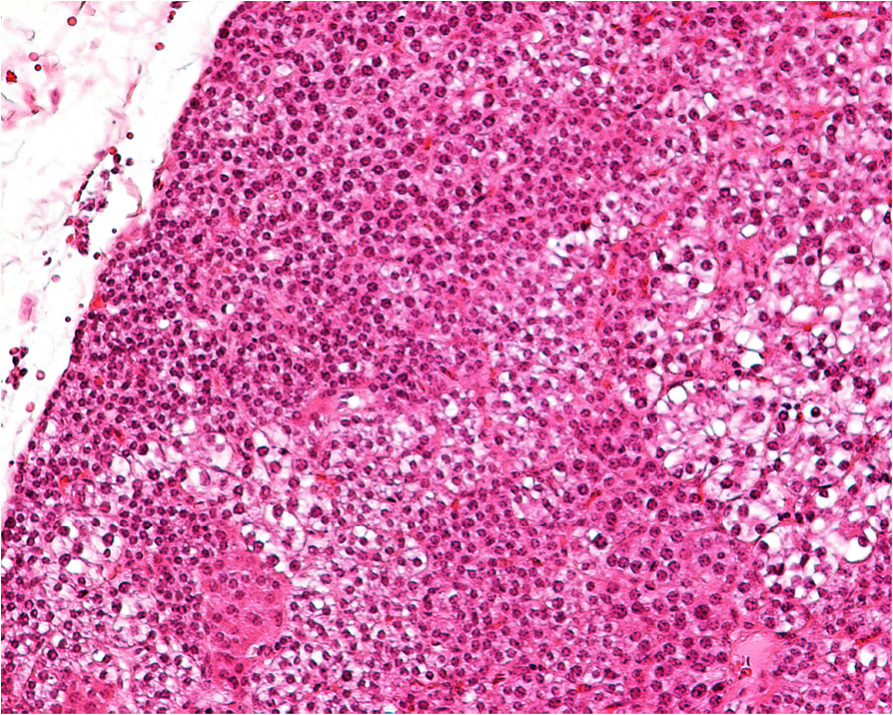
Microscopy appearance of parathyroid adenoma composed of a mixture of chief, oxyphil and clear cells in a diffuse growth pattern.

The pathology report confirmed the initial suspicion of a parathyroid adenoma and revealed a mixture of oxyphil, chief and clear cells (the latter as the dominant type) with the absence of intermediate fatty tissue (Figure 5).

The specimen was also sent for immunohistochemical examination for p-gp because the “reverse” scintigraphic tetrofosmin finding (positive early phase to negative delayed phase) combined with the size of the removed nodule (1 cm) was consistent with a parathyroid adenoma rich in p-gp. Unfortunately, immunohistochemical staining for p-gp cell expression was unable to be performed for technical reasons. However, shortly after the operation, the patient’s PTH levels began to gradually decrease and, two months later, her PTH level had returned to normal (13.8 pg/ml). This confirmed our initial hypothesis of a parathyroid adenoma.

## Discussion

The most commonly used radionuclide agent for the detection of parathyroid adenomas in clinical practice is ^99m^Tc- sestamibi. Sestamibi is a positively charged lipophilic isonitrile cationic derivative of technetium. It was originally used for myocardial perfusion imaging. With the dual-phase technique, sestamibi is initially located in the thyroid and parathyroid glands (early phase), and washes out of the thyroid more rapidly than out of the parathyroid, providing a greater parathyroid to thyroid uptake ratio (delayed phase) [5].

The precise mechanism of this differential uptake and retention in the thyroid and parathyroid glands remains unclear. One theory involves the size of the tumor and its blood flow in that small adenomas and hyperplastic glands are less likely to be visualized than larger ones; other theories involve the function of cellular mitochondrial content [6]. Some studies correlate the density of oxyphil cells in the adenoma with the tracer accumulation [7].

Although ^99m^Tc- sestamibi is the agent of choice, dual-phase scintigraphy with ^99m^Tc-tetrofosmin, has also been used to detect parathyroid adenomas. Both agents are lipophilic cationic complexes, actively taken up by the cell, indicating cellular viability. ^99m^Tc-tetrofosmin uptake mainly depends on mitochondrial potential, while ^99m^Tc- sestamibi uptake depends on both cell and mitochondrial membrane potential. This may explain the clinical observation of a higher thyroid wash-out rate using ^99m^Tc- sestamibi. On the other hand, wash-out rate of ^99m^Tc- tetrofosmin from parathyroid adenomas is slower. This slightly different mechanism of uptake and wash out explains why ^99m^Tc- sestamibi seems superior to ^99m^Tc-tetrofosmin in detecting parathyroid adenomas using the dual-phase technique. However, the results are comparable, and ^99m^Tc-tetrofosmin laboratory preparation is easier because sestamibi must be heated for 10 minutes before examination [3,8,9].

The standard scintigraphic pattern of a positive parathyroid adenoma using the dual- phase technique, either with sestamibi or tetrofosmin, is that of a focal area of increased radiopharmaceutical uptake in the thyroid bed or mediastinum (as ectopic tissue), well visualized and differentiated from the radioactivity of the thyroid gland, that either progressively increases over time (for instance on late acquisition) or shows fixed and intense uptake. The technique is based on the observation that the radiotracer, especially sestamibi, washes out more rapidly from the normal thyroid gland than from the abnormal parathyroid gland where it is retained for a longer period of time. This retention mainly depends on the abundance of oxyphilic cells rich in mitochondria, where the tracer is easily trapped intracellularly [5,10].

False positive results in parathyroid imaging mainly arise from a hyperfunctioning thyroid nodule, either a solitary or multinodular gland. For this reason, a complete examination of the thyroid with ultrasound, hormone level measurement, palpation, and even scintigraphy prior to parathyroid examination is necessary. Less common causes of false positive results include thyroid carcinoma, lymphoma, lymphadenopathy from inflammation or metastatic disease and sarcoidosis [5].

False negative results are strongly dependent on the size of the lesion. As the lesion becomes smaller, the likelihood of detection is even lower. Poor spatial system resolution and little tracer uptake by small parathyroid adenomas explain this phenomenon. Adenomas with relatively few oxyphilic cells may also be undetectable, because the lack of these mitochondria-rich cells, leads to lower uptake and faster washout of the tracer from the lesion. Another reason for false negative results is the expression of p-gp in parathyroid tissue [5,7,11].

P-gp is a 170 kD transmembrane protein encoded by the human multidrug resistance gene (MDR). High levels of p-gp are mainly found in epithelial cells and protect them against toxicity. Different substrates, such as chemotherapeutic drugs, enter the cell and bind reversely to p-gp on the cytoplasmic side of the cell membrane. P-gp, functioning as an efflux pump, transports them out of the cell through the mediation of adenosine 5΄- triphosphate. This mechanism explains why p-gp is frequently observed in several chemotherapeutic resistant cancer tissues. Positive p-gp expression exports chemotherapeutic drugs from cell targets, as does sestamibi. Because it shares physical and chemical characteristics with substances transported by p-gp, its concentration within an adenoma cell rich in p-gp is problematic, because of poor scintigraphic visualization or the absence of detection. Although there is evidence of low p-gp expression in abnormal parathyroid tissue, studies correlating sestamibi scintigraphy and p-gp expression seem controversial [12,13]. Other studies have concluded that both a small adenoma size and p-gp expression may limit the sensitivity of sestamibi imaging to localize parathyroid adenomas preoperatively [14]. Similar results have been obtained from studies using tetrofosmin as a radionuclide tracer [15].

In cases of adenomas rich in p-gp, the standard scintigraphic pattern reverses. Instead of a hot radioactive spot indicating the presence of the adenoma in both the early and delayed phase, well visualized and differentiated from the rest activity (especially on delayed imaging), a high concentration of the tracer within the adenoma may be detected only in the early phase, disappearing in the delay imaging. This “reverse pattern” provides strong evidence of a relatively enlarged parathyroid adenoma rich in p-gp, because the efflux p-gp pump facilitates the outcome of the tracer and its rapid wash out from the lesion. However, only biopsy and immunohistochemical staining can prove this.

According to the results of our study, the “reverse” scintigraphic tetrofosmin finding (positive early phase to negative delayed phase) combined with the size of the removed nodule (1 cm) supported the initial assumption regarding the presence of a parathyroid adenoma, probably rich in p-gp.

## Conclusion

^99m^Tc-sestamibi scintigraphy is the imaging technique of choice for the diagnosis of parathyroid adenomas because it has a sensitivity of up to 90%. Although, the gold standard for parathyroid adenoma diagnosis is still the scintigraphy with ^99m^Tc-sestamibi, both ^99m^Tc-tetrofosmin and ^99m^Tc-sestamibi are used for the diagnosis of parathyroid adenoma in the everyday clinical practice setting. We believe that dual-phase scintigraphy with ^99m^Tc-tetrofosmin, which is used for the detection of parathyroid adenomas, can play a leading role in the successful diagnosis of parathyroid ademonas because its uptake is related only to the mitochondrial potential, the preparation for examination preparation is easier and its diagnostic value is high. Moreover, the “reverse” scintigraphic tetrofosmin finding (positive early phase to negative delayed phase) combined with the size of the removed nodule (1 cm) supports the diagnosis of a parathyroid adenoma rich in p-gp. To conclude, we believe that dual-phase scintigraphy with ^99m^Tc-tetrofosmin can be a main, very useful, and effective part of parathyroid adenoma diagnosis in everyday clinical practice.

## Consent

Written informed consent was obtained from the patient for publication of this case report and accompanying images. A copy of the written consent is available for review by the Editor-in-Chief of this journal.

## Abbreviations

PTH: Parathormone; p-gp: p- glycoprotein; FT3: Free triiodothyronine; FT4: Free thyroxine; ALP: Alkaline phosphatase; MDR: Multidrug resistance gene.

## Competing interests

The authors declare that they have no competing interests.

## Authors’ contributions

ZA and KE carried out the ^99m^Tc-tetrofosmin Dual-phase Scintigraphy for the diagnosis of the parathyroid adenoma. PM, RK and NEA performed the parathyroidectomy. VT covered the anaeshegiological support during parathyroidectomy. GA and SE performed the pathological examination of the specimen. NEA and KE participated in the design of the study and analysed and interpreted the patient data regarding the parathyroid adenoma. PM, SC and ZA reviewed the manuscript and provided the final comments. All authors read and approved the final manuscript.
